# Data on characteristics of simplicia, phytoconstituents, and antioxidant activity of *Moringa oleifera* leaves ethanol extract

**DOI:** 10.1016/j.dib.2024.110425

**Published:** 2024-04-15

**Authors:** Fachruddin, Agik Suprayogi, Wasmen Manalu, Novriyandi Hanif, Huda Shalahuddin Darusman

**Affiliations:** a Postgraduate Student of School of Veterinary Medicine and Biomedical Sciences, IPB University, Bogor, Indonesia; b School of Veterinary Medicine and Biomedical Sciences, IPB University, Bogor, Indonesia; c Department of Chemistry, Faculty of Mathematics and Natural Sciences, IPB University, Bogor, Indonesia; d Tropical Biopharmaca Research Center, IPB University, Bogor, Indonesia; e Department of Biology, Faculty of Science and Technology, Institut Teknologi dan Kesehatan Avicenna, Kendari, Indonesia; f Primate Research Center, IPB University, Bogor, Indonesia

**Keywords:** *Moringa oleifera* leaves, Characteristics of simplicia, Phytoconstituents, LC-MS/MS, Antioxidant

## Abstract

The article reports data on the chemical and pharmacological characteristics of *Moringa* oleifera simplicia and ethanol extract consisting of water content, acid-insoluble ash content, microbial contamination, heavy metal contamination, phytochemical analysis, TLC analysis, chemical profiling using LC-MS/MS, and the antioxidant activity. The *M. oleifera* leaves simplicia meet quality standards, except for ash content that exceeds the specified standards. Qualitative phytochemistry indicates that the ethanol extract of *M. oleifera* leaves contains flavonoids, tannins, and steroids. In total of 39 phytoconstituents were tentatively identified; the top 10 active compounds with the highest relative abundance percentage (%) are 4-undecylbenzenesulfonic acid (4,83), apigetrin (3,34), quercetin-3β-D-glucoside (3,28), D-(-)-quinic acid (1,69), corchorifatty acid F (1,52), 4-hydroxybenzaldehyde (1,47), isopropylmalic acid (1,17), 13(*S*)-HOTrE (1,08), astragalin (0,99), and D-(+)-phenyllactic acid (0,70). The ethanol extract of *M. oleifera* leaves contained a total phenolic content 7728,02 mg/kg, total flavonoids as quercetin content 1,19%, and antioxidant activity IC_50_ 1422,45 mg/kg.

Specifications Table

**Table:** 

Subject	Biology
Specific subject area	Medicinal Plants and Pharmacology
Data format	Raw and Analyzed Data
Type of data	Table, Figure, Chart, Chromatogram Figure
Data collection	Characterizations of *Moringa oleifera* leaves simplicia were using gravimetric analysis techniques, pour plate method and Inductively Coupled Plasma – Optical Emission Spectrometry (ICP OES). Qualitative phytochemical data for the ethanol extract of *M. oleifera* leaves were obtained through color visualization. The UHPLC Vanquish Tandem Q Exactive Plus Orbitrap HRMS Thermo Scientific method was used to identify the active compounds. The total phenolic and total flavonoid as quercetin content in the ethanol extract of *M. oleifera* leaves were tested using UV-vis spectrophotometer, while antioxidant activity was evaluated using the 2,2-diphenyl-1-picrylhydrazyl (DPPH) method.
Data source location	*M. oleifera* leaves collection: Kokalukuna district, Baubau city, Southeast Sulawesi. Geographical coordinates: 05^o^ 25’41.68” S 122^o^ 39’12.47” E or 51M 461611 939987. Analysis: TropBRC IPB University; Certification Agency and Integrated Laboratory IPB University; and Laboratory of Balai Pengujian Standar Instrumen Tanaman Rempah, Obat dan Aromatik (BSIP-TROA), Bogor, Indonesia.
Data accessibility	Repository name: Mendeley Data Data identification number: 10.17632/hgn5gwy8h3.2 Direct URL to data: https://data.mendeley.com/datasets/hgn5gwy8h3/2

## 1 Value of the Data

• The data of characteristics of *M. oleifera* leaves simplicia provide important information about the fundamental properties of the plant material including quality consistency, quality control, and the safety of *M. oleifera* leaves raw materials.
• The data of phytochemical screening and active compounds in *M. oleifera* leaves extract, supported by growing environments data, are beneficial for comparative analysis of the chemical profiles of *M. oleifera* grown in different geographical conditions and may be useful in the selection of raw materials for medicinal purposes.
• Phytoconstituents data obtanied by LC-MS/MS provide detailed information about specific chemical constituents and can be useful for enriching the chemical compound database of *M. oleifera* plants for further investigations.
• Antioxidant activity data strengthens the evidence of the pharmacological activity of *M. oleifera* extract which is important in addressing various diseases such as inflammatory diseases, diabetic, cardiovascular diseases, cancer, neurodegenerative diseases, and various other diseases related to free radicals.

## 2 Background

*Moringa oleifera* Lam. is one of the most popular and important natural herbal plants cultivated globally [1], including in Indonesia. *M. oleifera* has garnered significant attention due to the remarkable benefits found in all parts of the plant including the roots, stems, leaves, flowers, fruits, and seeds [2]. It is a plant with high nutraceutical and medicinal potential being exceptionally rich in essential nutrients and phytochemicals [3,4]. Extract of *M. oleifera* evince diverse pharmacological activities, like antimicrobial, antioxidant, wound healing, and other pharmacological activities [5]. The purpose of compiling this dataset is to provide a proposal of comprehensive information on the characteristics of simplicia, active compounds, and antioxidant activity of *M. oleifera* leaves ethanol extract.

Theoretically, characterizing simplicia, determining active compounds, and testing antioxidant activity are crucial for assessing the quality and safety of raw materials [6] in developing *M. oleifera* leaves-based products with health benefits. The information contained in this dataset can be utilized by researchers, scientists, and healthcare professionals interested in harnessing *M. oleifera* leaves for therapeutic purposes. This article could benefit previous studies by offering a complete dataset that can be used for further analysis or compared with similar research.

## 3 Data Description

**Fig. 1:**
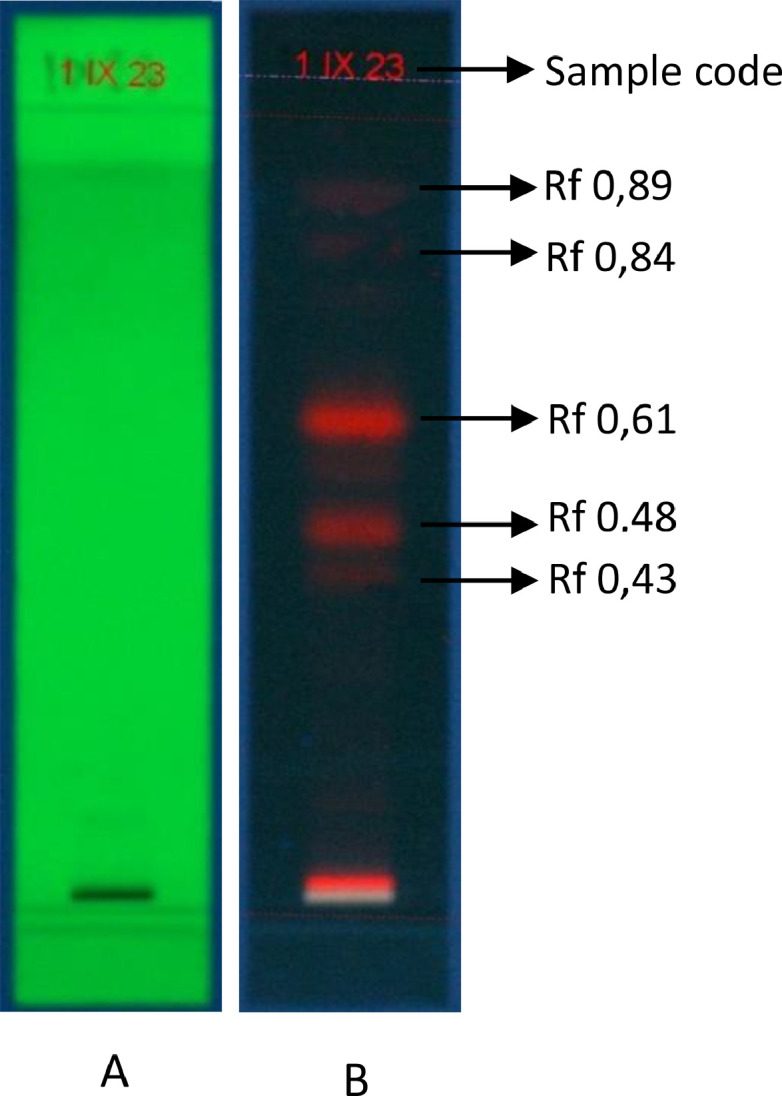
Chromatogram profile of *M. oleifera* leaves ethanol extract using thin layer chromatography (TLC). Mobile phase toluene:ethyl acetate (7:3), stationary phase silica gel F_254_. Captured the image in visible light (A) UV 254 nm, (B) UV 366 nm.

**Fig. 2:**
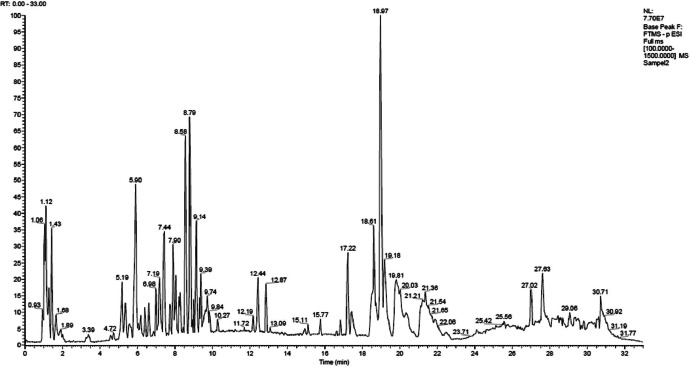
LC-MS/MS chromatogram of *M. oleifera* leaves ethanol extract.

**Fig. 3:**
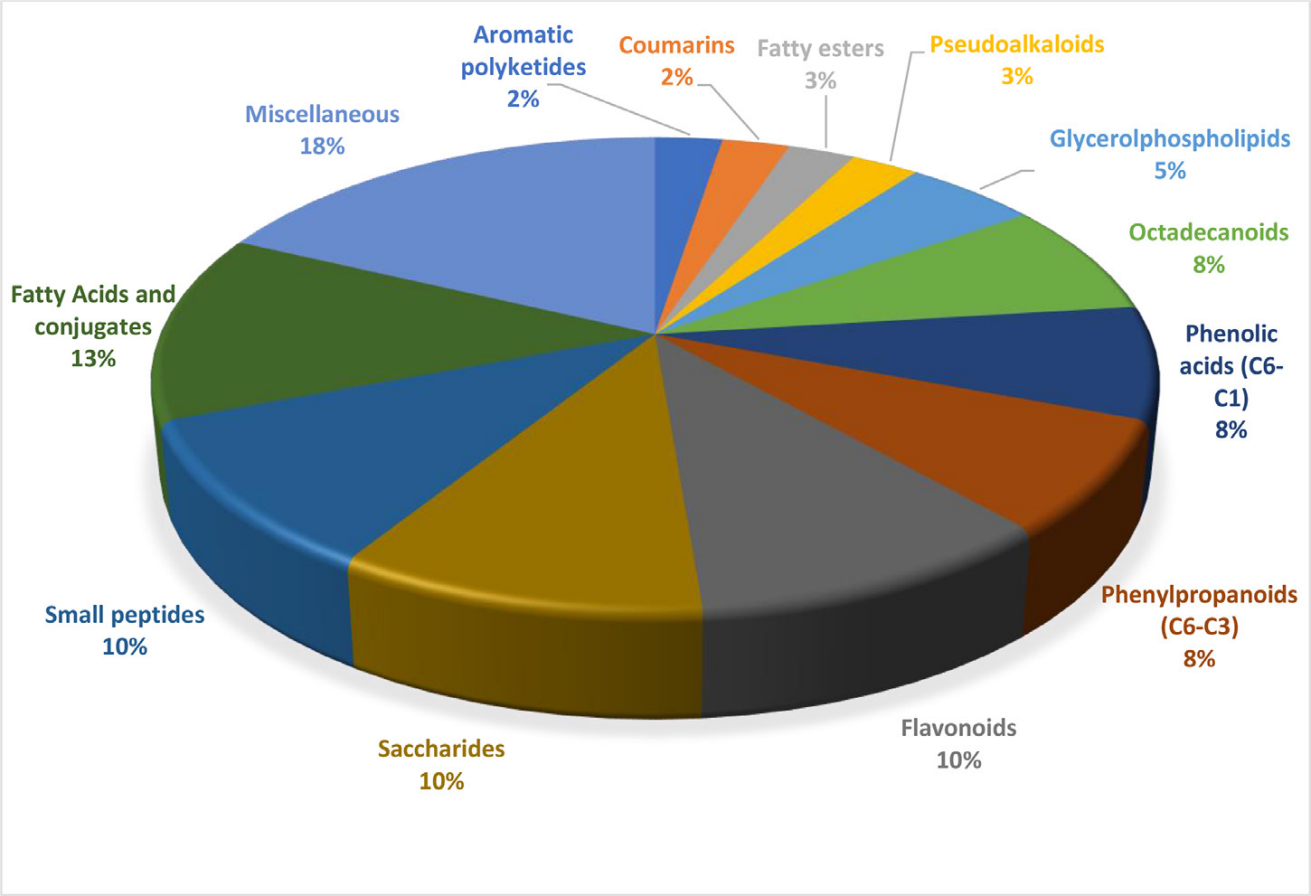
Pie chart indicating the percentage distribution of the compounds based on superclass in *M. oleifera* leaves ethanol extract.

The growing environments data of the *M. oleifera* plant collected as sample in this study were presented in Table 1, while the characteristics of *M. oleifera* leaves simplicia were shown in Table 2. The data of fresh weight to dry weight conversion of *M. oleifera* leaves, further from simplicia to the extract until obtaining a 70% ethanol extract yield were presented in Table 3. Qualitative phytochemical screening data and thin-layer chromatography profiles of the ethanol extract of *M. Oleifera* leaves were shown in Table 4 and Fig. 1, respectively. The chromatogram data of LC-MS/MS for the ethanol extract of *M. Oleifera* leaves and corresponding phytochemical were presented in Fig. 2 and Table 5, respectively, while Table 6 displayed data on the top 10 phytoconstituents with the highest relative abundance percentage. The distribution of the percentage of compound groups based on superclasses in sample was displayed in Fig. 3. Finally, the data of total phenolic content, total flavonoid as quercetin content, and antioxidant activity test with IC_50_ percentage as a parameter were depicted in Table 7.

**Table 1:** Growing environment of *M. oleifera* plant.

Environmental parameters	Result
Altitude (meters above sea level)	42
Air temperature (°C)	31
Air humidity (%)	76
Soil temperature (°C)	28
Soil moisture	Dry
Soil pH	6.5
Light intensity	Low

**Table 2:** The characteristics of *M. oleifera* leaves simplicia.

Parameters	Result	Reference [3]
Organoleptic Shape Smell Color	Powder Unique Green	
Water content (%)	9.68 ± 2,46	Not mentioned
Ash content (%)	10.85 ± 2,53	Not more than 7,55%
Acid insoluble ash content (%)	0.59 ± 0,04	Not more than 0,9%
Microbial contamination TPC (Total Plate Count) Mold/Yeast	2.5×10^4^ Negative	
Heavy metal contamination Pb Cd	Not detected Not detected

**Table 3:** Comparison of fresh weight, dry weight, percentage shrinkage of *M. oleifera* leaves, and yield of *M. oleifera* leaves ethanol extract.

Type of comparison	Result
Fresh weight (g)	1000 g
Dry weight (g)	216,54 g
Shrinkage percentage (%)	78,34 %
Simplicia weight (g)	500 g
70% ethanol volume (L)	15 L
Thick ethanol extract (g)	97,7 g ± 5,93
Yield percentage (%)	19,54 % ± 1,18

**Table 4:** Qualitative phytochemical screening of *M. oleifera* leaves ethanol extract.

Types of secondary metabolites	Result^a^^,^^b^
Flavonoid	++
Alkaloid Wagner Mayer Dragendorf	- - -
Tanin	++
Saponin	-
Quinon	-
Steroid	+
Triterpenoid	-

a qualitatively observed intensity degree of secondary metabolites. The more + sign was, the more content of secondary metabolites estimated.

b no corresponding secondary metabolites observed for - sign.

**Table 5:** Phytoconstituents tentatively identified based on retention time (RT) matching in the ethanol extracts of *M. oleifera* leaves by LC-MS/MS.

RT (Min)	Area Sampel	Name of compound	Mol. Formula	Mol. Weight
1,058	88802251,03	2-C-Methylerythritol 4-phosphate	C_5_H_13_O_7_P	216,039
1,065	89982335,1	Arabic acid	C_5_H_1_O_6_	166,047
1,066	276663923,2	Gluconic acid	C_6_H_12_O_7_	196,057
1,100	177062336,6	D-(-)-Quinic acid	C_7_H_12_O_6_	192,062
1,112	44054592,13	Hept-2-ulose	C_7_H_14_O_7_	210,073
1,114	61165180,28	Theophylline	C_7_H_8_N_4_O_2_	180,062
1,121	35928712,16	2,3,4,5-Tetrahydroxypentanal	C_5_H_10_O_5_	150,052
1,135	122074541,1	DL-Malic acid	C_4_H_6_O_5_	134,020
1,139	104430222,2	Citric acid	C_6_H_8_O_7_	192,026
1,455	69189632,42	4-Oxoproline	C_5_H_7_NO_3_	129,041
1,664	54781971,13	Methylmalonic acid	C_4_H_6_O_4_	118,025
3,383	35362106,04	Pantothenic acid	C_9_H_17_NO_5_	219,110
5,230	50791815,98	Neochlorogenic acid	C_16_H_18_O_9_	354,095
5,331	37270034,91	DL-Tryptophan	C_11_H_12_N_2_O_2_	204,089
5,365	83488328,93	Vanilloloside	C_14_H_20_O_8_	316,116
5,579	56778225,83	Bendiocarb	C_11_H_13_ NO_4_	223,084
5,833	123412571,6	Isopropylmalic acid	C_7_H_12_O_5_	176,067
6,034	38328748,89	Salicylic acid	C_7_H_6_O_3_	138,030
6,095	45062739,38	NP-000587	C_16_H_18_O_8_	338,100
6,185	49398908,43	Methyl α-aspartylphenylalaninate	C_14_H_18_N_2_O_5_	294,121
7,187	154639500	4-Hydroxybenzaldehyde	C_7_H_6_O_2_	122,035
7,381	92004149,39	NP-018609	C_16_H_30_O_10_	428,189
7,402	47912080,34	MUD	C_16_H_18_O_8_	338,100
7,742	65174571,76	(3R,5R)-1,3,5-Trihydroxy-4-{[(2E)-3-(4-hydroxy-3-methoxyphenyl)-2-propenoyl]oxy}cyclohexanecarboxylic acid	C_17_H_20_O_9_	368,111
8,057	110019839,7	Nimodipine	C_21_H_26_N_2_O_7_	418,174
8,237	73900976,15	D-(+)-Phenyllactic acid	C_9_H_10_O_3_	166,062
8,295	77306701,69	4-(9H-β-Carbolin-1-yl)-1,2,4-butanetriol	C_15_H_16_N_2_O_3_	272,116
8,564	350463808,1	Apigetrin	C_21_H_20_O_10_	432,105
8,804	343662266,2	Quercetin-3β-D-glucoside	C_21_H_20_O_12_	464,095
9,392	103607399,6	Astragalin	C_21_H_20_O_11_	448,100
9,858	59025245,09	Luteolin 7-O-malonylglucoside	C_24_H_22_O_14_	534,101
12,437	159145810,6	Corchorifatty acid F	C_18_H_32_O_5_	328,225
16,724	50476826,72	13(*S*)-HpOTrE	C_18_H_30_O_4_	310,214
17,343	39694644,14	Ascorbyl palmitate	C_22_H_38_O_7_	414,261
18,575	506172473,5	4-Undecylbenzenesulfonic acid	C_17_H_28_O_3_S	312,175
18,711	42793109,96	13(*S*)-HOTrE	C_18_H_30_O_3_	294,219
20,747	65065882,83	1-Oleoyl-lysophosphatidic acid	C_21_H_41_O_7_P	436,258
21,788	53758398,97	1-Heptadecanoyl-sn-glycero-3-phosphate	C_20_H_41_O_7_P	424,259
24,105	37937679,37	16-Hydroxyhexadecanoic acid	C_16_H_32_O_3_	272,235

**Table 6:** The top 10 phytoconstituents and their chemical structure with the highest relative abundance percentage (%).

RT (Min)	Relative abundance (%)	Name of compound	Mol. Formula	Mol. weight	Chemical structure
18,575	4,83	4-Undecylbenzenesulfonic acid	C_17_H_28_O_3_S	312,175	
8,564	3,34	Apigetrin	C_21_H_20_O_10_	432,105	
8,804	3,28	Quercetin-3β-D-glucoside	C_21_H_20_O_12_	464,095	
1,100	1,69	D-(-)-Quinic acid	C_7_H_12_O_6_	192,062	
12,437	1,52	Corchorifatty acid F	C_18_H_32_O_5_	328,225	
7,187	1,47	4-Hydroxybenzaldehyde	C_7_H_6_O_2_	122,035	
5,833	1,17	Isopropylmalic acid	C_7_H_12_O_5_	176,067	
18,711	1,08	13(S)-HOTrE	C_18_H_30_O_3_	294,219	
9,392	0,99	Astragalin	C_21_H_20_O_11_	448,100	
8,237	0,70	D(+)-Phenyllactic acid	C_9_H_10_O_3_	166,062

**Table 7:** Total phenolic content, total flavonoids as quercetin content, and antioxidant activity (IC_50_) in *M. oleifera* leaves ethanol extract.

Total phenolic	Total flavonoids as quercetin	Antioxidant activity (IC_50_)
7728,02 mg/Kg ± 84,45	1,19% ± 0,01	1422,45 mg/Kg ± 176,09

## 4 Experimental Design, Materials and Methods

### 4.1 Collection of plant material and preparation

The fresh leaves of *M. oleifera* were gathered from the Kokalukuna district, Baubau city, Southeast Sulawesi, with geographical coordinates 05° 25′41.68" S 122° 39′12.47" E or 51M 461611 939987. Before the collection, environmental parameters were measured at the moringa plant's growing location, including altitude (altimeter EXA tools), air temperature (mercury thermometer), air humidity (thermometer-hygrometer Power Star Apps), soil temperature, soil moisture, soil pH, and light intensity (all use soil survey instrument 4 in 1). Harvesting was conducted in the morning, in mid-April 2023. The collected leaves comprised a mixture of young and mature leaves. After washing with running tap water, the *M. oleifera* leaves were placed in a large food tray covered with thin black fabric and dried under sunlight. The dried leaves were ground using an electric blender and sieved through an 80-mesh to obtain powdered simplicia. The simplicia was characterized based on specific parameters (organoleptic test) and non-specific parameters (water content, ash content, acid-insoluble ash content, microbial contamination, and heavy metal contamination) [7]. Water content, ash content, and acid-insoluble ash content were analyzed using gravimetric analysis techniques, while microbial contamination and heavy metal contamination were evaluated using the pour plate method and ICP OES, respectively.

### 4.2 Plant sample extraction, phytochemical screening, and thin-layer chromatography

The dryed powdered leaves of *M. oleifera* were macerated with 70 ethanol, with the ratio 1:10 (ratio of simplicia powder and solvent). Duration of maceration based on the depiction in method described by Vongsak *et al*. [8]. Phytochemical screening test of extract conducted in accordance with Syahputra *et al*. [9], while profile chromatogram of TLC performed using a stationary phase of silica gel 60 F_254_ and a mobile phase of toluene:ethyl acetate (7:3), as set forth by Bata *et al*. [10].

### 4.3 Identification of phytoconstituents by liquid chromatography tandem mass spectrometry (LC-MS/MS) analysis

The qualitative analysis made reference to method developed by Umar *et al*. [11], with slight modifications. 5 mg of the sample was dissolved in 1 mL of MeOH, then filtered through a 0.2 µm PTFE membrane. Phytoconstituents identification was performed in a Vanquish Flex UHPLC-Q Exactive Plus Orbitrap High-Resolution Mass Spectrometer using Accucore C18, 100×2.1 mm, 1.5 µm (ThermoScientific) as the separation column. The source of MS ionization used was electrospray ionization (ESI) and Q-Orbitrap was used as the mass analyzer. The collision energy used for fragmentation was 18, 35, and 53 eV. Other conditions were as follows: spray voltage 3.8 kV, a capillary temperature of about 320°C, sheath gas and auxiliary gas flow rates of 15 and 3 mL/min, respectively.

The flow rate from the delivery system was adjusted at 0.2 mL/min, the autosampler temperature was maintained at 30°C, and the sample injection volume was 2 µL. The mobile phase consisted of (A) H2O + 0.1% formic acid and (B) acetonitrile + 0.1% formic acid. A linear gradient elution program was applied as follows: 0–1 min (5% B), 1–25 min (5–95% B), 25–28 min (95% B), 28–33 min (5% B). The total run time was 33 min, with relative abundance 0–100 and MS full-scan type (100–1500 m/z) in negative ionization mode.

### 4.4 Determination of total phenolic, total flavonoid, and antioxidant activity

The measurement of total phenolic content, total flavonoid content, and antioxidant activity evaluation of *M. oleifera* leaves ethanol extract was conducted with alluded to Sulastri *et al*. [12], with some adjustments. The total phenolic content and total flavonoid as quercetin content were tested using a spectrophotometer, while antioxidant activity was evaluated using the 2,2-diphenyl-1-picrylhydrazyl (DPPH) method.

## Limitations

Not applicable.

## Ethics Statement

We affirm that the current work excludes human subjects, animal experiments, or data from social media platforms.

## CRediT authorship contribution statement

**Fachruddin:** Investigation, Methodology, Writing – original draft. **Agik Suprayogi:** Conceptualization, Writing – review & editing. **Wasmen Manalu:** Conceptualization, Writing – review & editing. **Novriyandi Hanif:** Methodology, Writing – review & editing. **Huda Shalahuddin Darusman:** Conceptualization, Methodology.

## Data Availability

Data on chemical and pharmacological characteristics of simplicia and ethanol extract of Moringa oleifera leaves (Original data) (Mendeley Data).

## References

[1] Paikra B.K., Dhongade H.K.J., Gidwani B. (2017). Phytochemistry and pharmacology of *Moringa oleifera* Lam. J. Pharmacopunct..

[2] Oyeyinka A.T., Oyeyinka S.A. (2018). *Moringa oleifera* as a food fortificant: recent trends and prospects. J. Saudi Soc. Agric. Sci..

[3] Anzano A. (2021). *Moringa oleifera* Lam: a phytochemical and pharmacological overview. Horticulturae.

[4] Hodas F., Zorzenon M.R.T., Milani P.G. (2021). *Moringa oleifera* potential as a functional food and a natural food additive: a biochemical approach. An. Acad. Bras. Cienc..

[5] Pareek A. (2023). *Moringa oleifera*: an updated comprehensive review of its pharmacological activities, ethnomedicinal, phytopharmaceutical formulation, clinical, phytochemical, and toxicological aspects. Int. J. Mol. Sci..

[6] Utami N.F., Elya B., Hayun, Kusmardi (2022). Measurement of quality non-specific and specific-parameters of 70% ethanol extract and simplicia from cascara coffee robusta (*Coffea canephora* L.) and its potency as antioxidant. IOP Conf. Ser. Earth Environ. Sci..

[7] R. Depkes, “Parameter Standar Umum Ekstrak Tumbuhan Obat,” 2000.

[8] Vongsak B., Sithisarn P., Mangmool S., Thongpraditchote S., Wongkrajang Y., Gritsanapan W. (2013). Maximizing total phenolics, total flavonoids contents and antioxidant activity of *Moringa oleifera* leaf extract by the appropriate extraction method. Ind. Crops Prod..

[9] Syahputra R.A., Sutiani A., Silitonga P.M., Rani Z., Kudadiri A. (2021). Extraction and phytochemical screening of ethanol extract and simplicia of Moringa Leaf (*Moringa oleifera* Lam.) from Sidikalang, North Sumatera. Int. J. Sci. Technol. Manag..

[10] Bata M.H.C., Wijaya S., Setiawan H.K. (2018). Standarisasi Simplisia Kering Daun Kelor (*Moringa oleifera*) Dari Tiga Daerah Berbeda. J. Pharm. Sci. Pr..

[11] Umar A.H., Ratnadewi D., Rafi M., Sulistyaningsih Y.C. (2021). Untargeted metabolomics analysis using FTIR and UHPLC-Q-orbitrap HRMS of two curculigo species and evaluation of their antioxidant and α-glucosidase inhibitory activities. Metabolites.

[12] Sulastri E. (2018). Total phenolic, total flavonoid, quercetin content and antioxidant activity of standardized extract of *Moringa* oleifera *leaf* from regions with different elevation. Pharmacogn. J..

